# De novo assembly and analysis of *Polygonatum cyrtonema* Hua and identification of genes involved in polysaccharide and saponin biosynthesis

**DOI:** 10.1186/s12864-022-08421-y

**Published:** 2022-03-10

**Authors:** Dandan Li, Qing Wang, Songshu Chen, Hongchang Liu, Keqin Pan, Jinling Li, Chunli Luo, Hualei Wang

**Affiliations:** 1 Agronomy College, Guizhou University, Huaxi, Guiyang, Guizhou 550025 P. R. China; 2 Guizhou Key Laboratory of Propagation and Cultivation on Medicinal Plants, Huaxi, Guiyang, Guizhou 550025 P. R. China; 3 Construction Service Center of Wudang District Agricultural Science and Technology Zone, Wudang, Guiyang, Guizhou 550018 P. R. China

**Keywords:** *Polygonatum cyrtonema* Hua, Transcriptome, Polysaccharides, Saponins, Biosynthesis

## Abstract

**Background:**

The investigation of molecular mechanisms involved in polysaccharides and saponin metabolism is critical for genetic engineering of *Polygonatum cyrtonema* Hua to raise major active ingredient content. Up to now, the transcript sequences are available for different tissues of *P. cyrtonema*, a wide range scanning about temporal transcript at different ages’ rhizomes was still absent in *P. cyrtonema*.

**Results:**

Transcriptome sequencing for rhizomes at different ages was performed. Sixty-two thousand six hundred thirty-five unigenes were generated by assembling transcripts from all samples. A total of 89 unigenes encoding key enzymes involved in polysaccharide biosynthesis and 56 unigenes encoding key enzymes involved in saponin biosynthesis. The content of total polysaccharide and total saponin was positively correlated with the expression patterns of mannose-6-phosphate isomerase (*MPI*), GDP-L-fucose synthase (*TSTA3*), UDP-apiose/xylose synthase (*AXS*), UDP-glucose 6-dehydrogenase (*UGDH*), Hydroxymethylglutaryl CoA synthase (*HMGS*), Mevalonate kinase (*MVK*), 2-C-methyl-D-erythritol 2,4-cyclodiphosphate synthase (*ispF*), (E)-4-hydroxy-3-methylbut-2-enyl-diphosphate synthase (*ispG*), 4-hydroxy-3-methylbut-2-enyl diphosphate reductase (*ispH*), Farnesyl diphosphate synthase (*FPPS*). Finally, a number of key genes were selected and quantitative real-time PCR were performed to validate the transcriptome analysis results.

**Conclusions:**

These results create the link between polysaccharides and saponin biosynthesis and gene expression, provide insight for underlying key active substances, and reveal novel candidate genes including TFs that are worth further exploration for their functions and values.

**Supplementary Information:**

The online version contains supplementary material available at 10.1186/s12864-022-08421-y.

## Background

*Polygonatum cyrtonema* Hua (Asparagaceae) is a renowned traditional Chinese herb, and is also an edible plant. It has been widely applied for the treatment of many diseases such as dizziness, coughs et al. [1]. In Chinese Pharmacopoeia, “*polygonati rhizoma*” is often prescribed as the dried rhizome of *Polygonatum cyrtonema* Hua, *Polygonatum kingianum* Coll. et Hemsl and *Polygonatum* sibiricum Red [2]. A variety of medicinal effective ingredients have been isolated from “*polygonati rhizoma*” including Polysaccharides, saponins, flavonoids et al., and these effective ingredients exhibit a variety of vital pharmacological activities such as antioxidant, immunomodulatory, and anti-inflammatory et al. [3–5]. The previous research demonstrated the content of these effective ingredients including total polysaccharides and total saponins in *P. cyrtonema* plants changes with growth environment, cultivation technique, and growth years [6, 7]. This is of great significance for recognizing the biosynthesis and metabolism of polysaccharides and saponins.

Previously, in *P. cyrtonema*, researchers have revealed that polysaccharides were made of rhamnose, galactose, arabinose, mannose, glucose and fructose [6]. Partial researches verified that, in plant polysaccharides biosynthesis process, β-fructofuranosidase (sacA), hexokinase (HK), fructokinase (scrK), Phosphoglucomutase (PGM) is involved in the biosynthesis of NDP sugars [7–13]. Subsequently, abundant activated NDP-sugar precursors are added to polysaccharide residues promoting plant polysaccharides formation by a series of glycosyltransferase (GT) reactions [14]. In addition, some researches have revealed triterpene saponins biosynthesis pathway, and the number and expression level of partial key enzyme genes vary with the plant species [15–17].

A large volume of transcriptome, proteome and metabolomic have been executed in the post-genomic era [18]. Particularly, cause of the exact quantification of gene expression when lacking a reference genome, transcriptome sequencing (RNA-Seq) has been verified as the most useful, cost-effective technique for the research of metabolic pathways and function gene identification of effective ingredients [19].

In this study, we conducted a comprehensive analysis of the transcriptomes for different growth years rhizome of *P. cyrtonema* and identified plentiful candidate genes related to polysaccharide and triterpene saponins biosynthesis. The quality of our dataset was verified through quantitative real-time PCR (qRT-PCR). Our results provide a foundation for future researches that tackle the molecular mechanisms of polysaccharide and triterpene saponins biosynthesis in this species.

## Results

### Total polysaccharide content of *P. cyrtonema* samples

We extracted polysaccharides from the rhizomes with different growing years of *P. cyrtonema*. Results reveal that total polysaccharide content increased with the developmental years, the value was highest in three-year rhizomes (16.004%), subsequently decreased from three-year rhizomes to four-year rhizomes. The lowest value emerges in one-year rhizomes (7.76%) (Additional file 1: Fig. S1).

### Total saponin content of *P. cyrtonema* samples

Total saponin from the rhizomes with different growing years of *P. cyrtonema* were extracted. Results reveal that total saponin content increased with the developmental years, the value was highest in three-year rhizomes, subsequently decreased from three-year rhizomes to four-year rhizomes (Additional file 2: Fig. S2).

### Illumina sequencing and de novo transcriptome assembly

The results of sequencing data quality were presented in Additional file 3: Table S1. All these data sets were characterized by Q30 ≥ 94.79%. A total of 62,635 unigenes were generated. These unigenes had a mean length of 1007.11 bp and an N50 value of 1456 bp; 34.14% (21,388) and 63.47% (39,752) of these exceeded 1000 bp and 500 bp in length, respectively (Additional file 4: Fig. S3).

### Functional annotation and expression overview of unigenes

Out of the 62,635 unigenes identified in this analysis, 54.31, 38.90, 19.18, 31.14, 48.94, 35.40 and 31.66% unigenes were recorded as significant hits in the NR, SwissProt, KEGG, KOG, eggNOG, GO and Pfam databases, respectively (Table 1). Out of the 34,020 unigenes annotated in the NR database, 59.23, 7.53, 6.03, and 27.21% were mapped to the genes of *Asparagus officinalis* (Liliaceae), *Elaeis guineensis* (Arecaceae), *Phoenix dactylifera* (Palmae), and others, respectively (Additional file 5: Fig. S4). A total of 18,283 of these unigenes were then matched with one or more GO terms and comprise 50 functional groups (Additional file 6: Fig. S5). We found that ‘cellular process’ and ‘metabolic process’ were the most abundant categories within biological processes, while within the molecular function term, ‘binding’ and ‘catalytic activity’ were the most abundant.

**Table 1:** Summary of *P. cyrtonema* unigenes annotated in seven public databases

Database	Number annotated	Annotated unigene ratio (%)
NR	34,020	54.31
SwissProt	24,367	38.90
KEGG	12,015	19.18
KOG	19,503	31.14
eggNOG	30,651	48.94
GO	22,170	35.40
Pfam	19,833	31.66

**Fig. 1:**
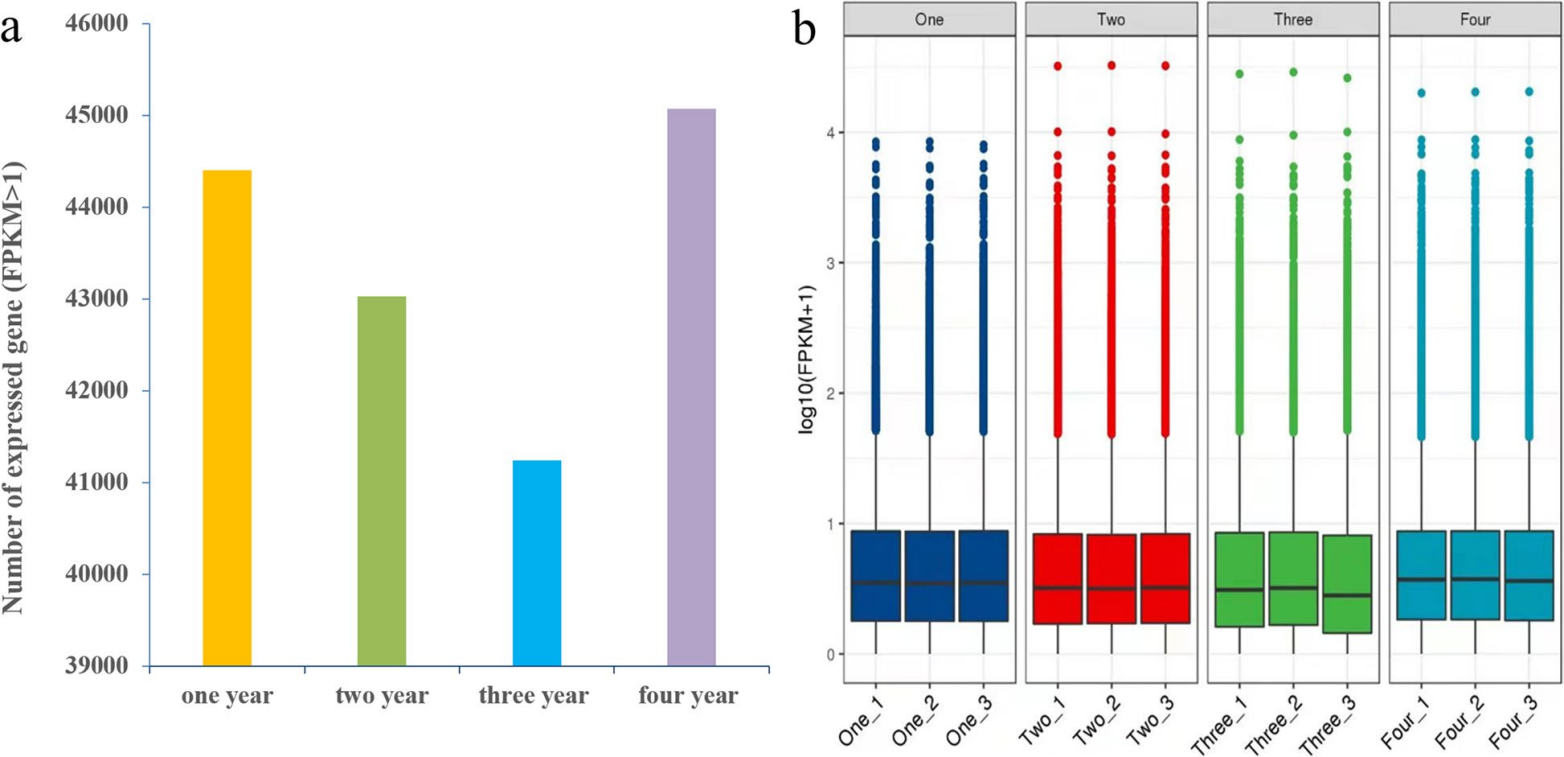
Expression profiles of genes in different years’s rhizome tissues of *P. cyrtonema.***a** Distributions of average expressed unigenes (FPKM> 1) in the four samples. **b** Bloxplot of unigenes expressed in the four samples with three duplications, respectively. X-axis represents the different year’s rhizome tissues, and Y- axis shows the log_10_ (FPKM+ 1) values. Signifcant test of 12 samples is performed using multi-independent sample krukal-wallis test

Unigenes with FPKM> 1 was counted in each tissue. The results of this comparison showed that average 44,403, 43,030, 41,245 and 45,076 unigenes were expressed in one-year, two-year, three-year, four-year rhizome samples, respectively (Fig. 1a). Gene expression level was highest in four-year rhizome compared with other rhizomes (Fig. 1b).

### Identification of genes involved in polysaccharide biosynthesis

**Fig. 2:**
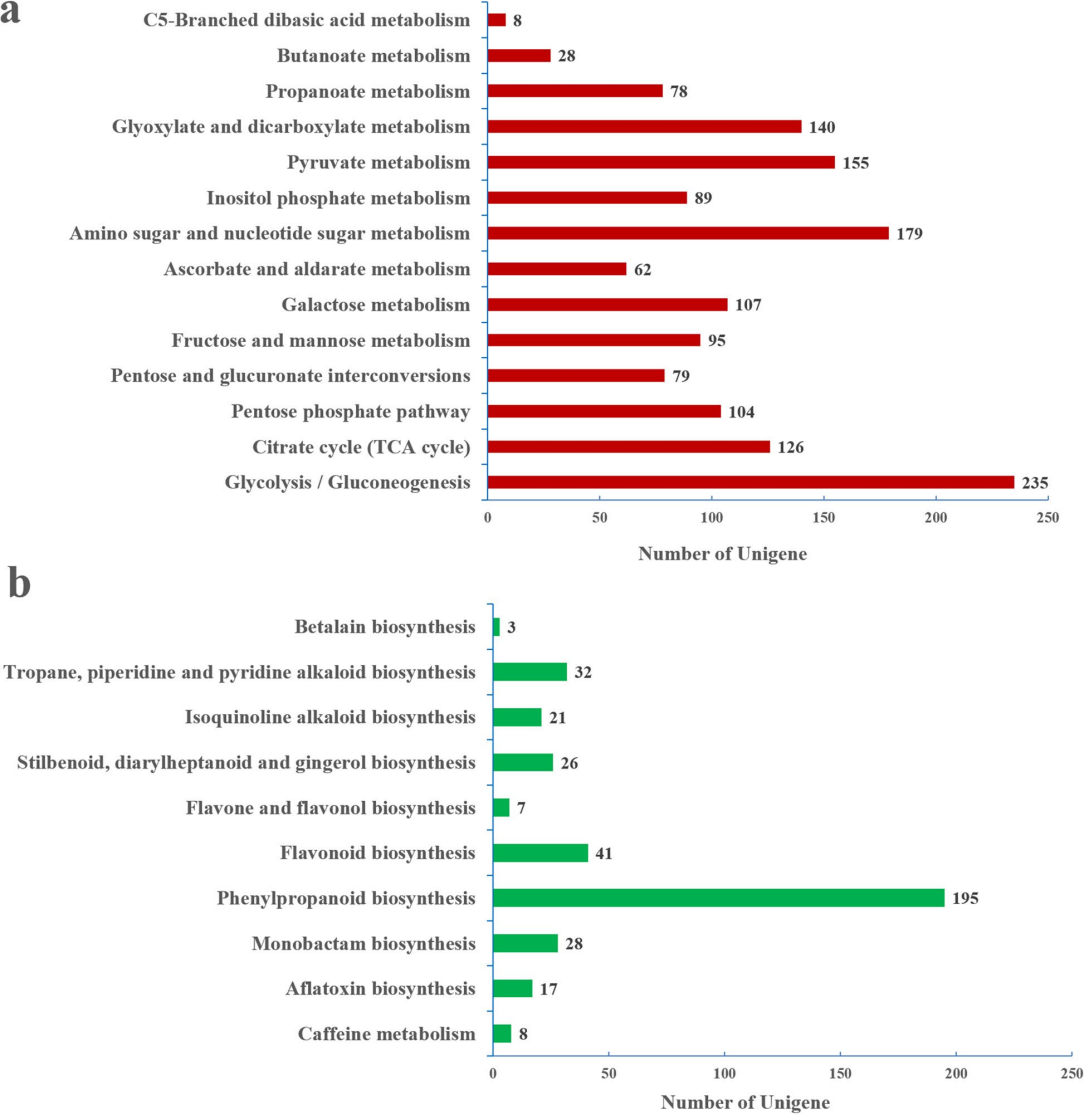
KEGG annotation of *P. cyrtonema* unigenes. **a** Pathway classifications for carbohydrate metabolism. **b** Pathway classification for the biosynthesis of other secondary metabolites

To comprehend the most noteworthy biological processes in *P. cyrtonema*, a total of 12,015 unigenes were annotated and allocated to 125 pathways (20 subcategories) (Additional file 7: Fig. S6 and Additional file 8: Table S2). The ‘carbohydrate metabolism’ subcategory involved in14 pathways with the largest number of unigenes (235) included glycolysis/gluconeogenesis metabolism. Besides, 588 unigenes were corresponding in polysaccharide biosynthesis pathways, including amino and nucleotide sugar metabolism, fructose and mannose metabolism, glycolysis/gluconeogenesis, and pentose and glucuronate interconversions (Fig. 2a). A total of 10 pathways were allocated to the biosynthesis of other secondary metabolites and the amplest unigenes within this set were marked within the phenylpropanoid biosynthesis pathway (Fig. 2b).

**Fig. 3:**
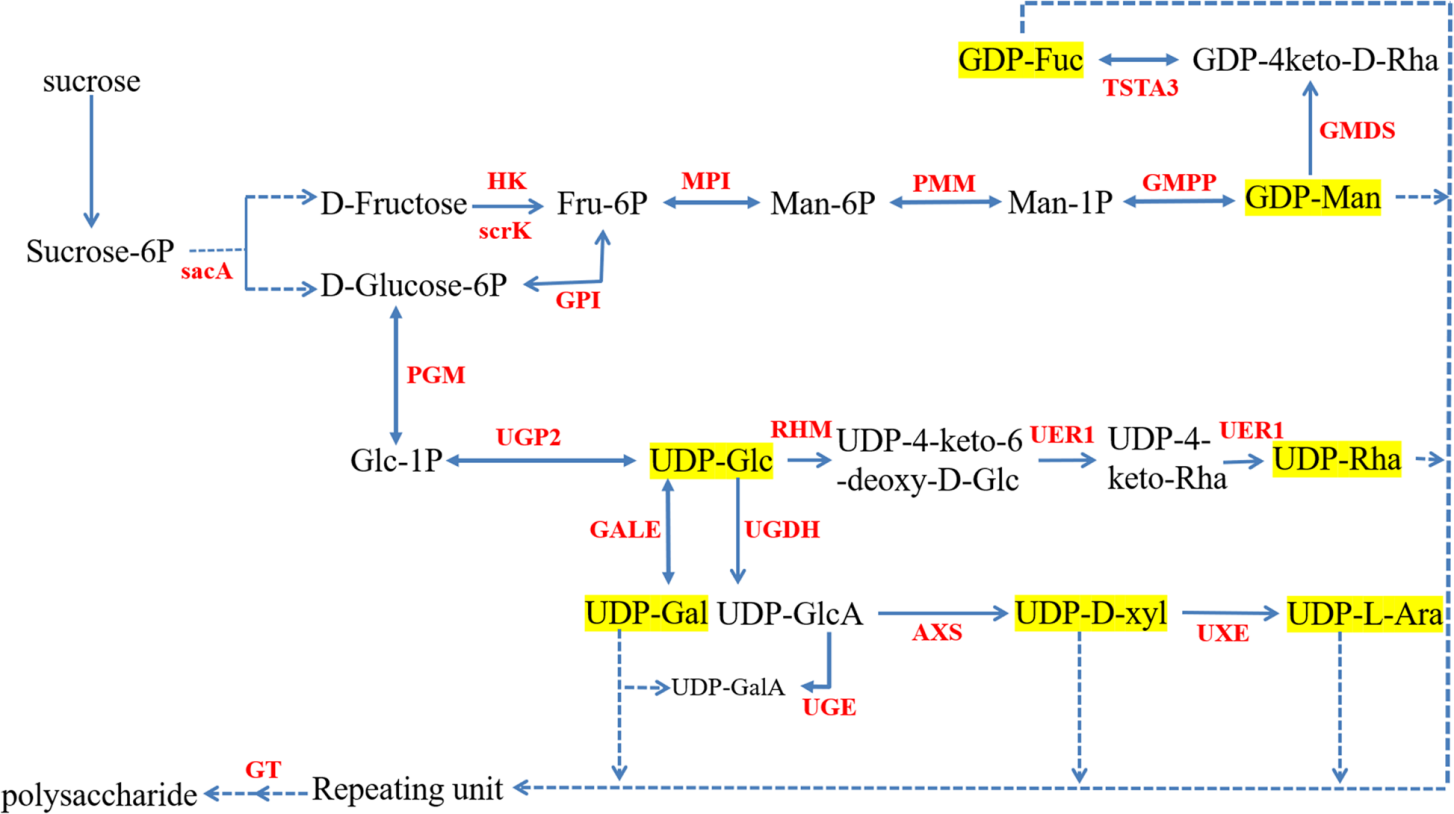
Proposed pathways for polysaccharide biosynthesis in *P. cyrtonema*. Note: Arrows with solid lines represent the identified enzymatic reactions, and arrows with dashed lines represent multiple enzymatic reactions through multiple steps. Activated monosaccharide units, marked in black with yellow background and the enzymes, marked in red

**Fig. 4:**
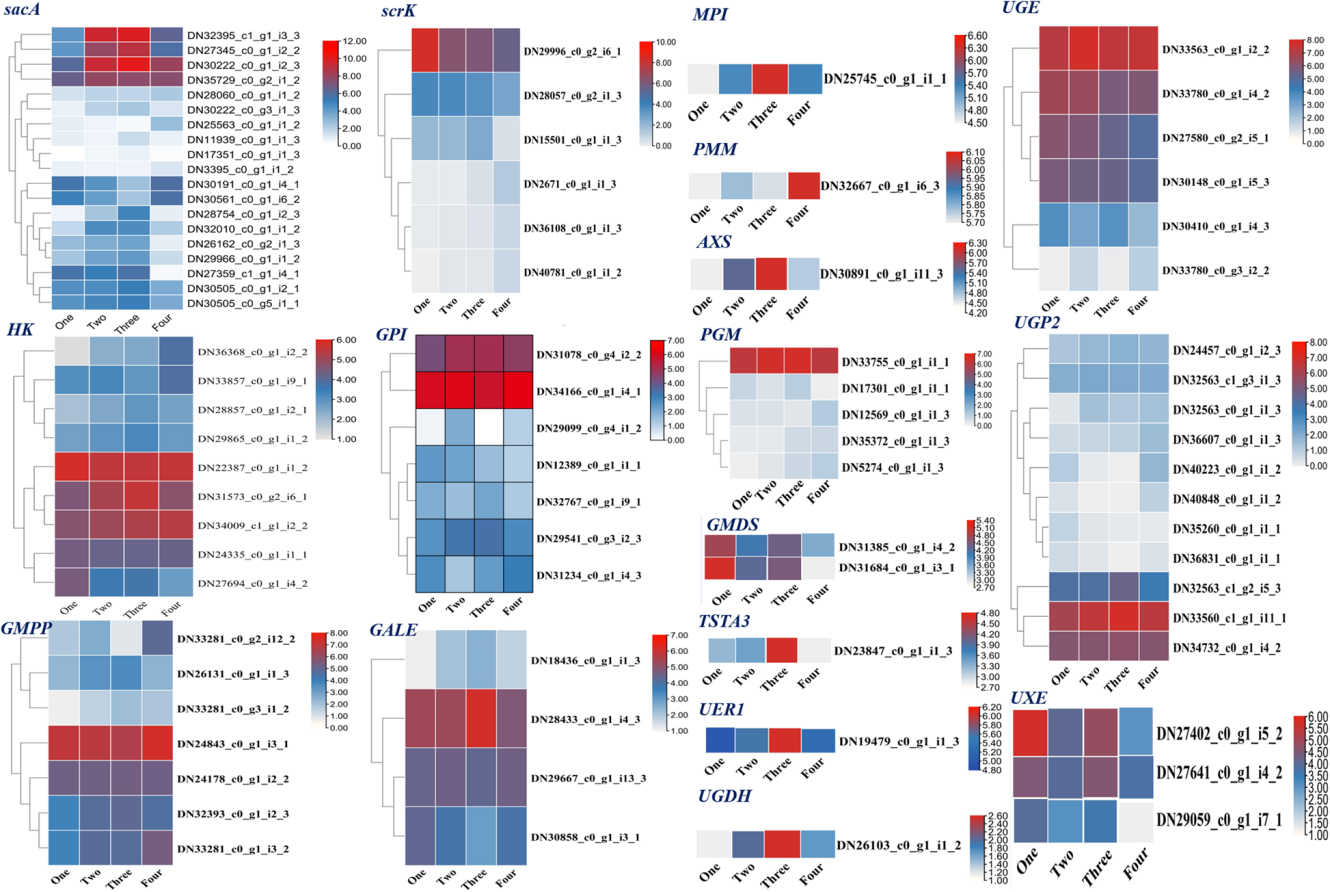
Total expression levels of unigenes encoding enzymes involved in polysaccharide biosynthesis. Note: The columns one, two, three and four represent one-year, two-year, three-year and four-year rhizome samples, respectively. Red, blue and grey represent high, medium and low expression levels, respectively

In order to enhance our understanding of polysaccharide biosynthesis, we annotated 274 unigenes involved in amino and nucleotide sugar metabolism (Ko00520) and fructose and mannose metabolism (Ko00051) pathways based on the KEGG database. A total of 89 unigenes encoding key enzymes, including 3,5-epimerase-4-reductase (UER1), UDP-glucose 4-epimerase (GALE), UDP-arabinose 4-epimerase (UXE) et al. (Table 2). These data enabled the identification of genes encoding enzymes involved in polysaccharide biosynthesis using the FPKM approach (Figs. 3 and 4).

**Table 2:** Number of unigenes encoding key enzymes involved in polysaccharide biosynthesis in *P. cyrtonema*

Enzyme name	EC number	Unigene number
beta-fructofuranosidase (sacA)	3.2.1.26	19
hexokinase (HK)	2.7.1.1	9
fructokinase (scrK)	2.7.1.4	6
mannose-6-phosphate isomerase (MPI)	5.3.1.8	1
phosphomannomutase (PMM)	5.4.2.8	1
mannose-1-phosphate guanylyltransferase (GMPP)	2.7.7.13	7
GDPmannose 4,6-dehydratase (GMDS)	4.2.1.47	2
GDP-L-fucose synthase (TSTA3)	1.1.1.271	1
glucose-6-phosphate isomerase (GPI)	5.3.1.9	7
phosphoglucomutase (PGM)	5.4.2.2	5
UTP--glucose-1-phosphate uridylyltransferase (UGP2)	2.7.7.9	11
UDP-glucose 4-epimeras (GALE)	5.1.3.2	4
UDP-glucose 4-epimerase (UGE)	5.1.3.6	6
UDP-glucose 6-dehydrogenase (UGDH)	1.1.1.22	1
UDP-apiose/xylose synthase (AXS)	–	1
UDP-arabinose 4-epimerase (UXE)	5.1.3.5	3
UDP-glucose 4,6-dehydratase (RHM)	4.2.1.76	4
3,5-epimerase/4-reductase (UER1)	5.1.3.-1.1.1.-	1

### Identification of genes involved in saponins biosynthesis

**Fig. 5:**
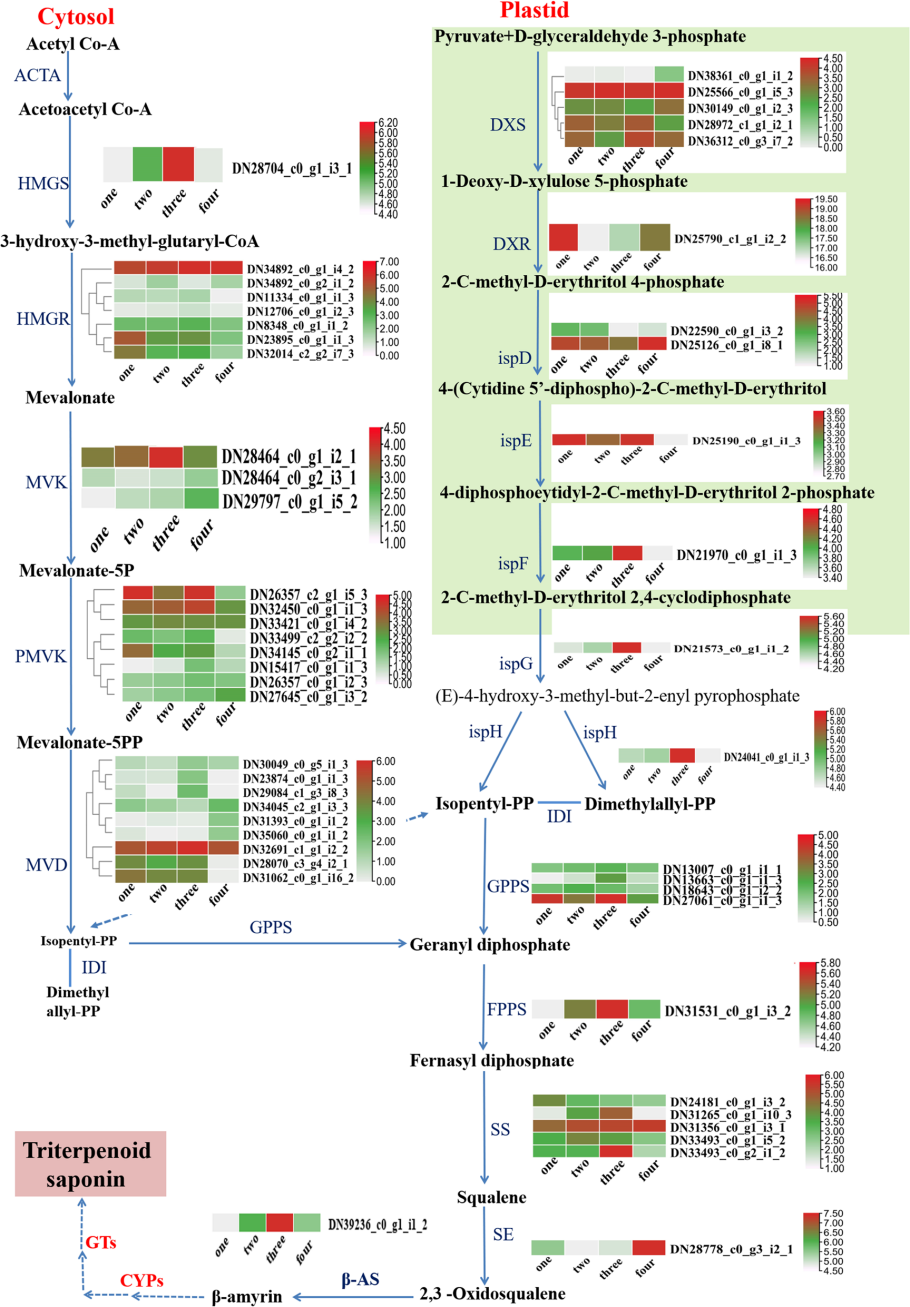
Total expression levels of unigenes encoding enzymes involved in triterpene saponin biosynthesis. Note: The columns one, two, three and four represent one-year, two-year, three-year and four-year rhizome samples, respectively. Red, green and grey represent high, medium and low expression levels, respectively

In order to enhance our understanding of triterpene saponins biosynthesis, we also annotated unigenes involved in terpenoid backbone biosynthesis (Ko00900) and carotenoid biosynthesis (Ko00906) pathways based on the KEGG database. A total of 56 unigenes encoding key enzymes, including hydroxymethylglutaryl CoA synthase (HMGS), mevalonate kinase (MVK), 1-deoxy-D-xylulose-5-phosphate synthase (DXS), 1-deoxy-D-xylulose-5-phosphate reductoisomerase (DXR), geranylgeranyl diphosphate synthase (GGPS), squalene synthase (SS) et al. (Table 3). These data enabled the identification of genes encoding enzymes involved in triterpene saponins biosynthesis using the FPKM approach (Fig. 5).

**Table 3:** Number of unigenes encoding key enzymes involved in triterpene saponin biosynthesis in *P. cyrtonema*

Enzyme name	EC number	Unigene number
Acetyl-CoA acetyl transferase (AATC)	2.3.1.9	0
Hydroxymethylglutaryl CoA synthase (HMGS)	2.3.3.10	1
3-hydroxy-3-methylglutaryl-coenzyme A reductase (HMGR)	1.1.1.34	7
Mevalonate kinase (MVK)	2.7.1.36	3
Phosphomevalonate kinase (PMVK)	2.7.4.2	8
Mevalonate diphosphosphate decarboxylase (MVD)	4.1.1.33	9
1-deoxy-D-xylulose-5-phosphate synthase (DXS)	2.2.1.7	5
1-deoxy-D-xylulose-5-phosphate reductoisomerase (DXR)	1.1.1.267	1
2-C-methyl-D-erythritol4-phosphate cytidylyltransferase (ispD)	2.7.7.60	2
4-diphosphocytidyl-2-C-methyl-D-erythritol kinase (ispE)	2.7.1.148	1
2-C-methyl-D-erythritol 2,4-cyclodiphosphate synthase (ispF)	4.6.1.12	1
(E)-4-hydroxy-3-methylbut-2-enyl-diphosphate synthas (ispG)	1.17.7.1	1
4-hydroxy-3-methylbut-2-enyl diphosphate reductase (ispH)	1.17.7.4	1
Isopentenyl-diphosphate Delta-isomerase (IDI)	5.3.3.2	1
Geranyl diphosphate synthase (GPS)	2.5.1.1	3
Geranylgeranyl diphosphate synthase (GGPS)	2.5.1.29	5
Farnesyl diphosphate synthase (FPPS)	2.5.1.10	1
Squalene synthase (SS)	2.5.1.21	5
Squalene epoxidase (SE)	1.14.14.17	1

### Validation and expression analysis of genes encoding key enzymes

**Fig. 6:**
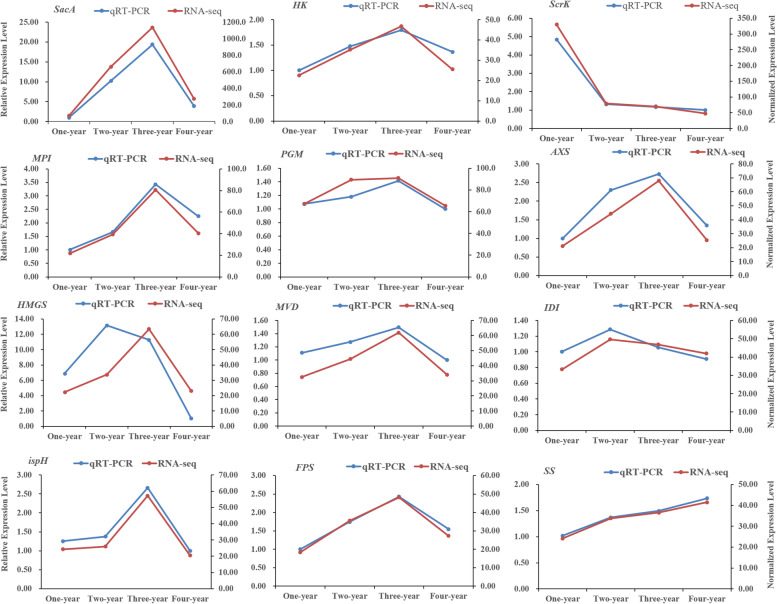
The expression levels of 12 genes at one-year, two-year, three-year and four-year rhizomes in *P. cyrtonema* for qRT-PCR and RNA-seq experiment (mean ± SD, *n* = 3)

To validated the reliability of transcriptome sequencing data, the expression levels of genes encoding beta-fructofuranosidase (*sacA*), fructokinase (*scrk*), mannose-6-phosphate isomerase (*MPI*), phosphoglucomutase (*PGM*), UDP-apiose/xylose synthase (*AXS*), hydroxymethylglutaryl CoA synthase (*HMGS*), mevalonate diphosphosphate decarboxylase (*MVD*), isopentenyl-diphosphate delta-isomerase (*IDI*), farnesyl diphosphate synthase (*FPPS*) and squalene synthase (*SS*) et al. were tested by qRT-PCR assays The results revealed the qRT-PCR data for these 16 genes were basically consistent with the RNA-Seq data (Fig. 6, the numerical values of error bar is presented in Additional file 9: Table S3). Generally, the above results revealed that our transcriptome data were reliable for genes temporal expression analysis during the rhizome developmental processes in *P. cyrtonema.*

### Identification of DEGs

**Fig. 7:**
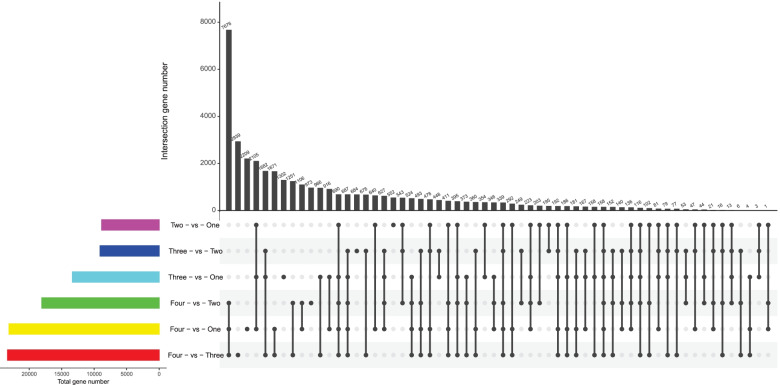
Venn diagram of differentially expressed genes (DEGs) among different *P. cyrtonema* rhizomes samples. Note: the abscissa on the left reflects the number of genes and the ordinate represent different comparison groups, the black dot is used to connect different regions to represent the common gene of different comparison groups and the number of these common genes is displayed by the bar graph on the right

DEGs were recognized in all different developmental rhizomes using FPKM values for unigenes. When one-year rhizomes were set as the control, and 8850, 13,361 and 23,107 different expressed genes (DEGs) (*p*-value< 0.05 and fold change> 1.5) were identified at two-year, three-year and four-year rhizomes, respectively. When two-year rhizomes were set as the control, a total of 9101 and 18,067 DEG were identified at three-year and four-year rhizomes, respectively. When three-year rhizomes were set as the control, a total of 23,332 DEGs were identified at four-year rhizomes (Fig. 7).

### Identification of TFs involved in the biosynthesis of polysaccharides, saponins and other secondary metabolites

A total of 1492 TFs were identified in transcriptome database of *P. cyrtonema.* Cause of the contents of polysaccharides and saponins all increase from one-year rhizomes to three-year rhizomes, one-year vs three-year contrast was analyzed, 245 TFs were up-regulated and 135 TFs were down-regulated (Table 4). The major TF families were identified in this analysis included AP2/ERF-ERF (107 unigenes), WRKY (106 unigenes), NAC (89 unigenes), bHLH (85 unigenes), C2H2 (84 unigenes), C3H (79 unigenes) and MYB-related (76 unigenes) groups.

**Table 4:** Type and number of transcription factors (TFs) of *P. cyrtonema*

TF_family	Number of unigenes	Up-regulated unigenes	Down-regulated unigenes
AP2/ERF-AP2	10	5	0
AP2/ERF-ERF	107	26	12
AP2/ERF-RAV	3	0	2
Alfin-like	4	0	0
B3	42	5	4
B3-ARF	19	1	0
BBR-BPC	5	0	0
BES1	8	1	0
C2C2-CO-like	6	2	0
C2C2-Dof	26	9	1
C2C2-GATA	24	5	3
C2C2-LSD	5	1	0
C2C2-YABBY	6	4	1
C2H2	84	13	6
C3H	79	8	1
CAMTA	2	0	0
CPP	4	0	0
CSD	3	0	0
DBB	4	0	0
DBP	3	1	0
E2F-DP	8	0	0
EIL	3	0	0
FAR1	64	2	5
GARP-ARR-B	7	1	0
GARP-G2-like	46	6	4
GRAS	33	8	1
GRF	11	4	1
GeBP	16	2	1
HB-BELL	12	4	0
HB-HD-ZIP	25	6	0
HB-KNOX	8	3	0
HB-PHD	2	0	0
HB-WOX	6	1	1
HB-other	23	1	1
HRT	1	0	0
HSF	35	6	8
LIM	3	0	0
LOB	17	3	0
MADS-M-type	23	0	0
MADS-MIKC	12	6	1
MYB	67	19	4
MYB-related	76	9	6
NAC	89	10	17
NF-X1	3	1	0
NF-YA	8	0	1
NF-YB	11	4	1
NF-YC	9	1	0
OFP	12	7	0
PLATZ	13	2	1
RWP-RK	8	0	0
S1Fa-like	4	0	0
SAP	1	0	1
SBP	22	7	1
SRS	5	0	0
STAT	1	0	0
TCP	24	7	1
TUB	21	1	0
Tify	25	2	3
Trihelix	25	1	2
ULT	1	0	0
VOZ	1	0	0
WRKY	106	5	31
Whirly	5	1	0
bHLH	85	18	9
bZIP	58	11	4
zf-HD	9	4	0
zn-clus	4	1	0

### SSR marker analysis

To develop SSR markers in *P. cyrtonema*, MISA software was applied to identify the SSRs sites among 62,635 unigenes. A total of 17,351 SSRs were identified (SSR > =1, 13,116 unigenes; SSR > =2, 3159 unigenes; compound SSRs, 1567 unigenes). In 17,357 SSRs founded the mono-nucleotide repeat motifs were the most abundant types (45.51%), followed by di-nucleotide (31.12%), tri-nucleotide (21.79%), hexa-nucleotide (0.67%), tetra-nucleotide (0.66%), and penta-nucleotide tandem repeats (0.25%, Table 5).

**Table 5:** Distribution of identified SSRs of *P. cyrtonema*

Motif	Repeat numbers								Total	%
	5	6	7	8	9	10	11	> 11		
Mono-	0	0	0	0	0	3838	1497	2562	7897	45.51
Di-	0	1769	1051	656	433	279	179	1033	5400	31.12
Tri-	2165	895	389	179	85	46	8	14	3781	21.79
Tetra-	83	24	6	1	0	0	0	0	114	0.66
Penta-	31	11	1	0	0	0	0	0	43	0.25
Hexa-	92	13	5	3	1	0	1	1	116	0.67
Total	2371	2712	1452	839	519	4163	1685	3610	17,351	100
%	13.66	15.63	8.37	4.84	2.99	23.99	9.71	20.81	100	

## Discussion

*P. cyrtonema* is a well-known medical and edible plant, and it has a variety of biology activities such as anti-aging, nourishing yin, anti-inflammatory and immunomodulatory et al. [3–5]. Although polysaccharides and saponins are the significant effective constituents, however, up to now, genomic data is still unknown and only a copy of transcriptome data without biological duplications for three tissues of *P. cyrtonema* is available [20], that is obviously inadequate for demonstrating the molecule mechanisms of active constituents’ biosynthesis such as polysaccharide and saponins. In this study, we obtained a more reliable and high-quality assembly result (unigenes with an average length of 1007.11 bp) than previous transcriptome data (mean length 710 bp) in *P. cyrtonema*, also enriches the types of gene expression data, and facilitate the selection of key candidate genes involved in polysaccharides and saponins biosynthesis, condense the number of candidate genes to be verified.

A large number of unigenes participated in polysaccharide and saponins biosynthesis were identified (Figs. 4 and 5). For polysaccharide biosynthesis pathway, the genes encoding MPI, AXS, TSTA3, UER1, GALE and UGDH enzymes were high expressed in three-year rhizomes compared with other-year rhizomes, and these gene expression pattern is consistent with the accumulation pattern of polysaccharide with the rhizome development from one-year to four-year (Fig. 4, Additional file 1: Fig. S1), while the genes encoding HK and scrk demonstrate opposite pattern of expression against the accumulation of polysaccharide. Similar phenomenon was also observed in previous researches [20–23]. We speculate that *MPI*, *AXS*, *TSTA3*, *UER1*, *GALE* and *UGDH* are underlying key enzyme genes play vital roles in regulating the polysaccharide content of *P. cyrtonema* rhizomes and HK, scrk are mainly participate in other pathway such as sugar signaling, carbohydrate metabolism et al. [24]. For saponin biosynthesis pathway, the genes encoding HMGS, MVK, ispF, ispG, ispH and FPPS enzymes were high expressed in three-year rhizomes, and these gene expression pattern is consistent with the accumulation pattern of total saponin with the rhizome development (Fig. 5, Additional file 2: Fig. S2). It seems that MEP and MVA pathway all participated in the saponin biosynthesis [15, 17].

Plenty of TFs have been isolated and verified participating in a diversity of plant biological processes including biosynthesis of polysaccharides, saponins and other secondary metabolism processes. In our results, A total of 380 candidate TFs were allocated to the AP2/ERF-ERF, WRKY, NAC, bHLH, C2H2, C3H and MYB-related families; these TFs probably play roles in regulating polysaccharide and saponins biosynthesis. Previous Researches revealed *GubHLH3* positively regulates soyasaponin biosynthetic genes in *Glycyrrhiza uralensis* [25] and the *bHLH* transcription factors *TSAR1* and *TSAR2* regulate triterpene saponin biosynthesis in *Medicago truncatula* [26], A total of 85 candidate unigenes encoding *bHLH* TFs were identified, of which 18 and 9 were up-regulated in the three-year rhizome compared with other-year rhizome, respectively (Table 4). Over-expression of *AtMYB46* gene can enhance mannan content of hemicellulose polysaccharides [27]. A total of 67 candidate unigenes encoding MYB TFs were recognized, of which 19 and 4 were up-regulated in the three-year rhizome, respectively. These up-regulated unigenes are vital for subsequent studies aimed at exploring the regulation of polysaccharide and saponins biosynthesis in *P. cyrtonema*. The characterization of these unigenes will be beneficial for realizing the molecular mechanisms underlying polysaccharide and saponin biosynthesis.

## Materials and methods

### Ethics statement

Experimental materials were harvested across China, but the field studies did not involve endangered or protected species. This study was conducted at the in Guizhou Key Laboratory of Propagation and Cultivation on Medicinal Plants in Southwest China, Guiyang, China.

### Plant material

*P. cyrtonema* rhizomes were collected from the teaching and experimental farm of Guizhou University (116°40′E, 39°96′N) and identified as *Polygonatum cyrtonema* Hua (Asparagaceae) by Professor Hualei Wang (Guizhou University of Agronomy College). All plant samples were cleaned, removed fibrous root, dried on filter paper and then instantly frozen in liquid nitrogen.

### Extraction and determination of total polysaccharide and saponins

Total polysaccharides were extracted and detected from freeze-dried rhizomes samples of *P. cyrtonema* as described in Chinese Pharmacopoeia [2]. Total saponins were extracted and detected by colorimetry. Three repetitions have been done and a statistical analysis been performed by SPSS 22.0 software.

### Total RNA extraction, cDNA library construction and sequencing

The total RNA of one-year, two-year, three-year, four-year rhizomes with three biological replicates isolated using an E.Z.N.A. Plant RNA Kit (Omega Biotech Co. Ltd., USA) (Additional file 10: Table S4). RNA quality including integrity and concentration were evaluated using Huang et al.’s method [28]. The RNA-Seq libraries were generated using TruSeq Stranded mRNA Library Prep Kit for Illumina (San Diego, CA, USA). Then qualified libraries were sequenced using an Illumina HiSeqTM 4000 platform (Ouyi biology, Shanghai, China).

### De novo assembly and unigene function annotation

Low quality reads were removed before data analysis and high-quality clean reads were used to assemble using Trinity software [29]. For the CDS sequences which had no hits in Blast, ESTScan was used for predicting [30]. According to sequence similarities, functional annotations for unigenes were executed and mapped to seven databases including NCBI non-redundant, Swiss-Prot, KEGG (Kyoto Encyclopedia of Genes and Genomes) protein databases, KOG database, eggNOG database, GO and Pfam database. In addition, GO functional annotations were also attained with Nr annotation using the Blast2GO (version 2.5.0) [31]. KEGG Orthology annotations were further conducted using BlastX algorithm against KEGG database.

### Differential expression analysis

The quantitative expression level of unigenes for four rhizomes with different growth years were subjected using Expression Analyzer and DisplayER software (EXPANDER) [32]. The abundance of corresponding unigene transcripts were determined by the FPKM method. We compared unigenes that display differences in expression level between two rhizomes (i.e., one-year rhizome vs. two-year rhizome) using DESeq Software [33]. The FDR ≤ 0.001 and the fold change (FC) ≥ 2 were identified as DEGs.

### Analysis of transcription factors (TFs)

For transcriptome data, in *P. cyrtonema*, the open reading frames (ORF) were determined by the getorf software [34]. Then we aligned these ORFs to all TF protein domains using the plant transcription factor database (PlnTFDB) via BLASTX (e-value≤1e^− 5^) [35].

### Real-time PCR (qRT-PCR) analysis

Total RNA was isolated from *P. cyrtonema* rhizome (one-year, two-year, three-year, four-year) using the E.Z.N.A. Total RNA Kit I (Omega, USA) and reverse-transcribed to cDNA with TaKaRa reverse transcription reagents (TaKaRa Bio, Dalian, China). The elongation factor 1-ɑ (*EF1ɑ,* TRINITY_DN27092_c0_g5_i1_1) genes were selected as endogenous references for normalization according to its expression level and stability in transcriptome data. Specific primers were designed by primer 3.0 (Additional file 11: Table S5). Real-time PCR was performed by QuantiNova Syb^r^ Green PCR kit (Qiagen). The results of the target gene relative to the reference gene were calculated by the 2^-ΔΔCt^ method [36]. Data are presented as the mean ± standard deviation (SD) of three reactions performed in different 96-well plates. The data were analyzed using CFX Manager™ v3.0.

## Conclusion

A comprehensive transcriptome analysis of one-year, two-year, three-year and four-year rhizome with three duplications in P. cyrtonema were executed and abundant genes and TFs related to polysaccharide and saponin biosynthesis and regulation were identified, respectively. In addition, adequate SSRs marker were founded in transcriptome data that provides a significant convenience for the identification of *P. cyrtonema* plant. We used qRT-PCR technology to validate the results of transcriptome sequence and our results play a vital role in illuminating the polysaccharide and saponin biosynthesis pathways and facilitate future researches involved in accumulation of secondary metabolism in *P. cyrtonema*.

## Supplementary Information

**Additional file 1: Figure S1.** Total polysaccharide content in tuber of *Polygonatum cyrtonema* Hua of different growing years.

**Additional file 2: Figure S2.** Total saponin content in tuber of *Polygonatum cyrtonema* Hua of different growing years.

**Additional file 3: Table S1.** The results of sequencing data quality.

**Additional file 4: Figure S3.** Sequence length distribution for *P. cyrtonema* transcriptome assembly.

**Additional file 5: Figure S4.** Species distribution annotated in the NR database for *P. cyrtonema*.

**Additional file 6: Figure S5.** GO function annotation of *P. cyrtonema* transcriptome.

**Additional file 7: Figure S6.** KEGG functional classifications of the annotated unigenes in *P. cyrtonema*.

**Additional file 8: Table S2.** KEGG annotation of all unigenes.

**Additional file 9: Table S3.** The numerical values of error bar.

**Additional file 10: Table S4.** RNA information of different samples.

**Additional file 11: Table S5.** Gene descriptions and primers used for qRT-PCR.

## Data Availability

The data that support the findings of this study are openly available in the NCBI Sequence Read Archive (SRA) under projects PRJNA755493.
