# Supplementary material for: De novo assembly and analysis of Polygonatum cyrtonema Hua and identification of genes involved in polysaccharide and saponin biosynthesis
Source: BMC Genomics. 2022 Mar 10;23:195. doi: 10.1186/s12864-022-08421-y (PMC8915509; doi:10.1186/s12864-022-08421-y)
Supplement: Supplementary file 1 — Additional file 1: Figure S1. Total polysaccharide content in tuber of Polygonatum cyrtonema Hua of different growing years. [file 12864_2022_8421_MOESM1_ESM.docx]

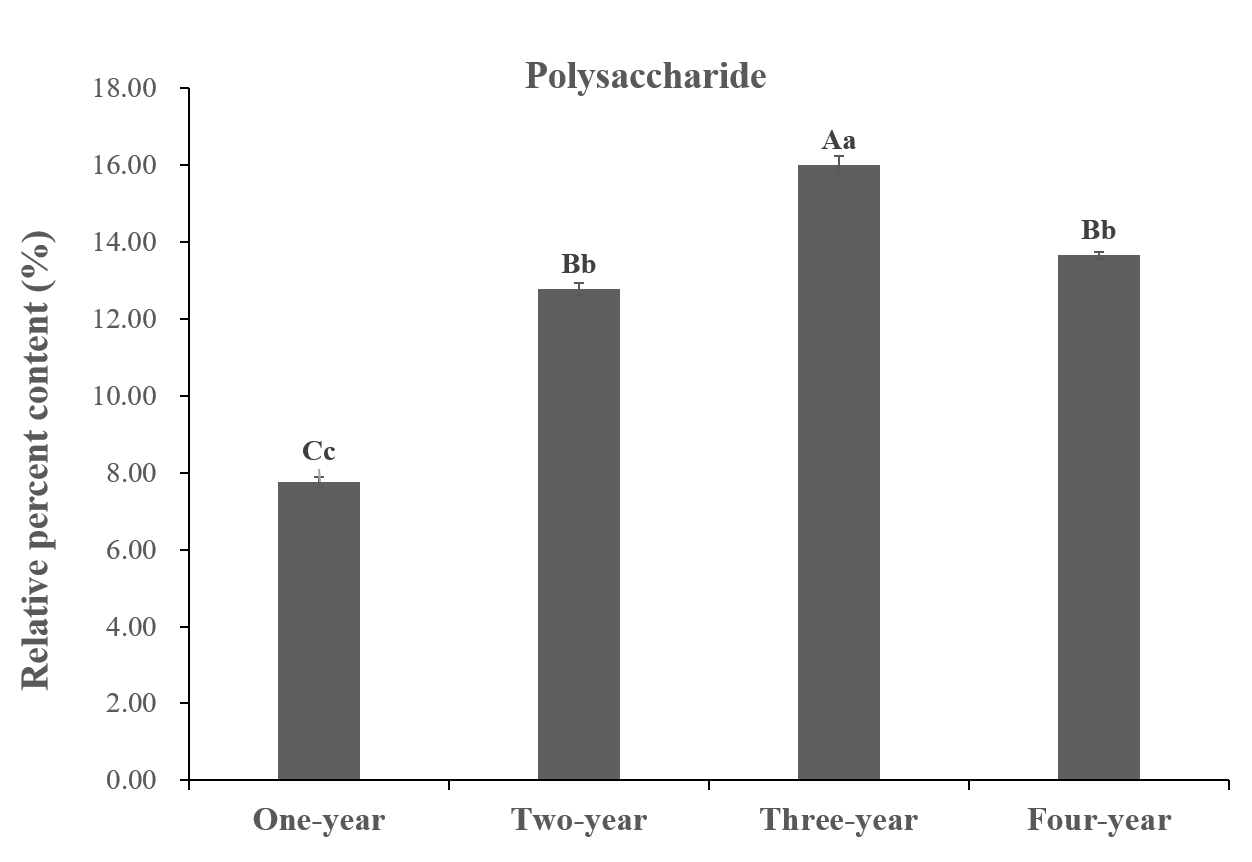


**Figure S1** **Total polysaccharide content in tuber of *Polygonatum cyrtonema* Hua of different growing years** **(mean ± SD, n=3).**

Note: The capital letters and lowercase letters indicate significance difference at *P<0.01* and *P<0.05*, respectively.
