# Supplementary material for: De novo assembly and analysis of Polygonatum cyrtonema Hua and identification of genes involved in polysaccharide and saponin biosynthesis
Source: BMC Genomics. 2022 Mar 10;23:195. doi: 10.1186/s12864-022-08421-y (PMC8915509; doi:10.1186/s12864-022-08421-y)
Supplement: Supplementary file 3 — Additional file 3: Table S1. The results of sequencing data quality. [file 12864_2022_8421_MOESM3_ESM.docx]

**Table S1** **The results of sequencing data quality.**

| **Sample** | **CleanBases** | **Clean Reads** | **Q30** | **GC** |
| --- | --- | --- | --- | --- |
| One-year-1 | 6.20 G | 42.92 M | 95.10% | 49.01% |
| One-year-2 | 6.61 G | 46.85 M | 94.97% | 48.94% |
| One-year-3 | 5.88 G | 40.97 M | 95.04% | 49.01% |
| Two-years-1 | 5.74 G | 40.12 M | 94.97% | 49.19% |
| Two-years-2 | 6.41 G | 45.92 M | 95.02% | 49.12% |
| Two-years-3 | 6.22 G | 43.52 M | 95.07% | 49.09% |
| Three-years-1 | 6.28 G | 43.93 M | 95.09% | 49.34% |
| Three-years-2 | 5.81 G | 40.53 M | 95.14% | 49.34% |
| Three-years-3 | 6.62 G | 46.25 M | 95.07% | 49.39% |
| Four-years-1 | 6.94 G | 48.81 M | 94.84% | 48.28% |
| Four-years-2 | 6.55 G | 45.74 M | 94.92% | 48.33% |
| Four-years-3 | 6.13 G | 42.80 M | 94.79% | 48.41% |
