# Supplementary figures and images for: De novo assembly and analysis of Polygonatum cyrtonema Hua and identification of genes involved in polysaccharide and saponin biosynthesis
Source: BMC Genomics. 2022 Mar 10;23:195. doi: 10.1186/s12864-022-08421-y (PMC8915509; doi:10.1186/s12864-022-08421-y)

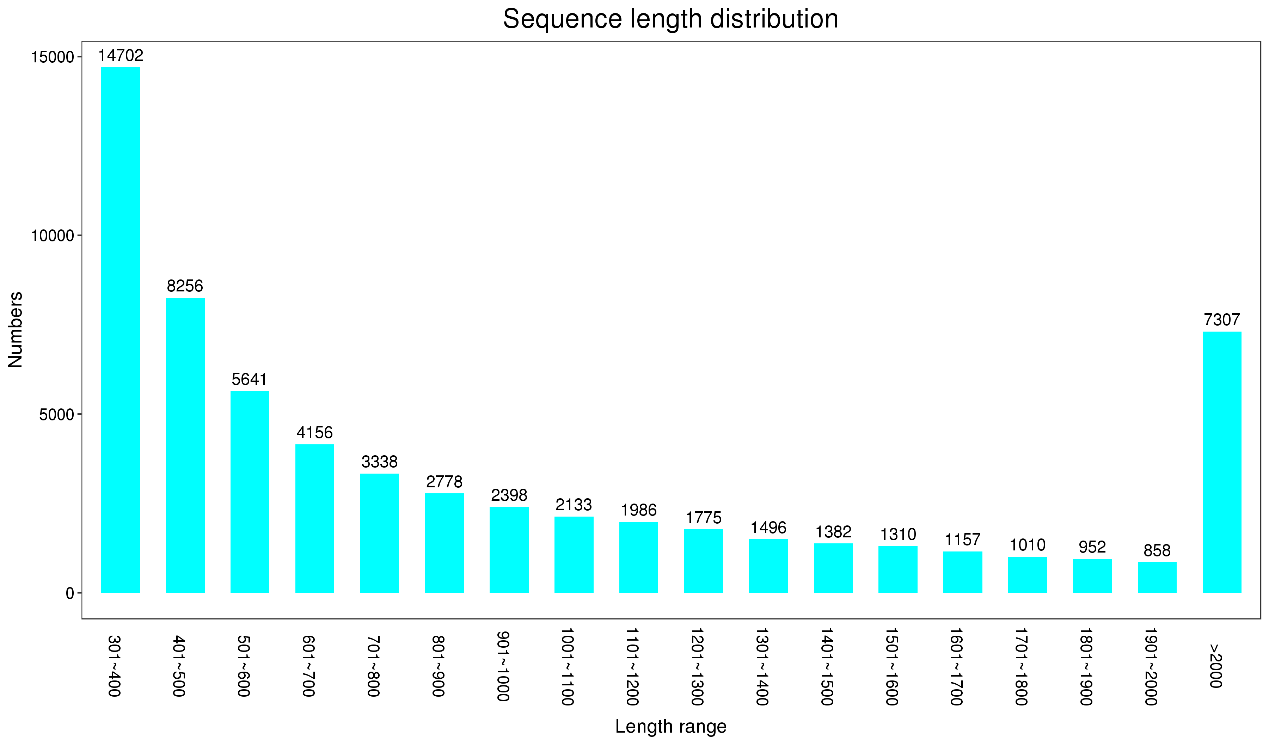


**Figure S3** **Sequence length distribution for *P. cyrtonema* transcriptome assembly.**

Supplement: Supplementary file 4 — Additional file 4: Figure S3. Sequence length distribution for P. cyrtonema transcriptome assembly. [file 12864_2022_8421_MOESM4_ESM.docx]

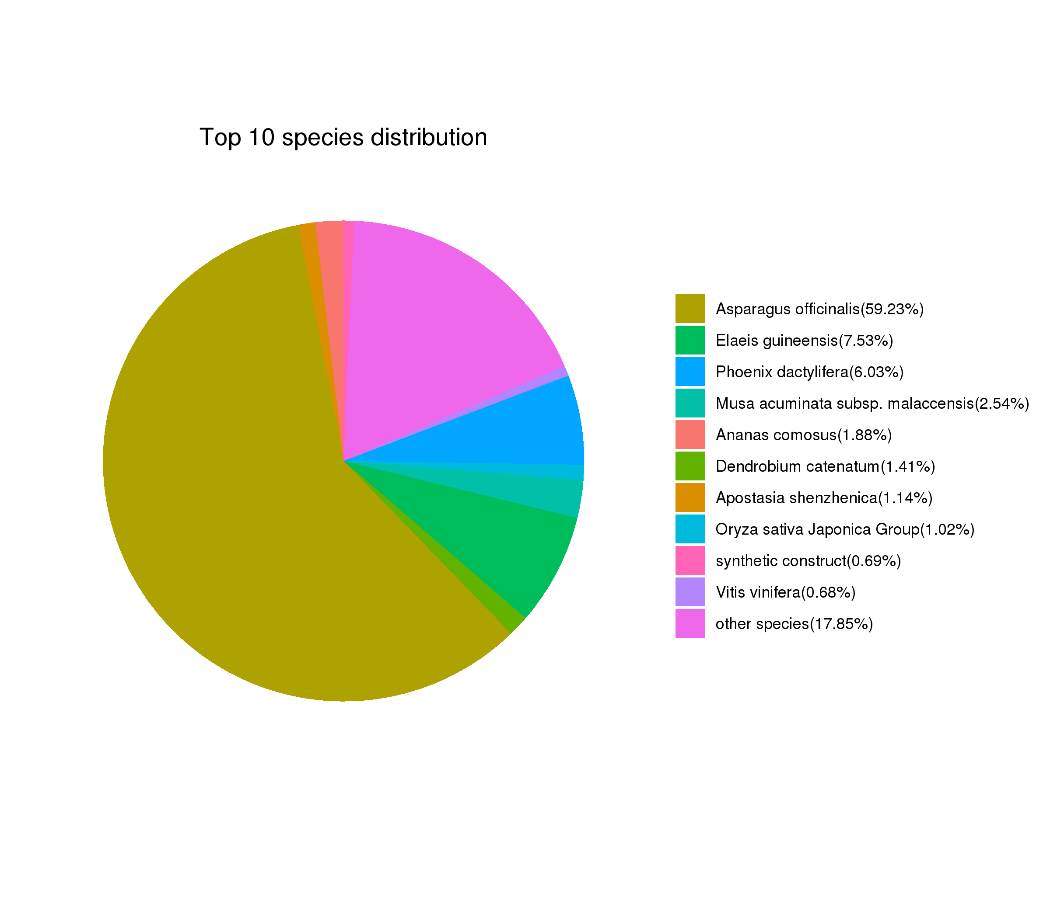


**Figure S4** **Species distribution annotated in the NR database for** ***P. cyrtonema.***

Supplement: Supplementary file 5 — Additional file 5: Figure S4. Species distribution annotated in the NR database for P. cyrtonema. [file 12864_2022_8421_MOESM5_ESM.docx]

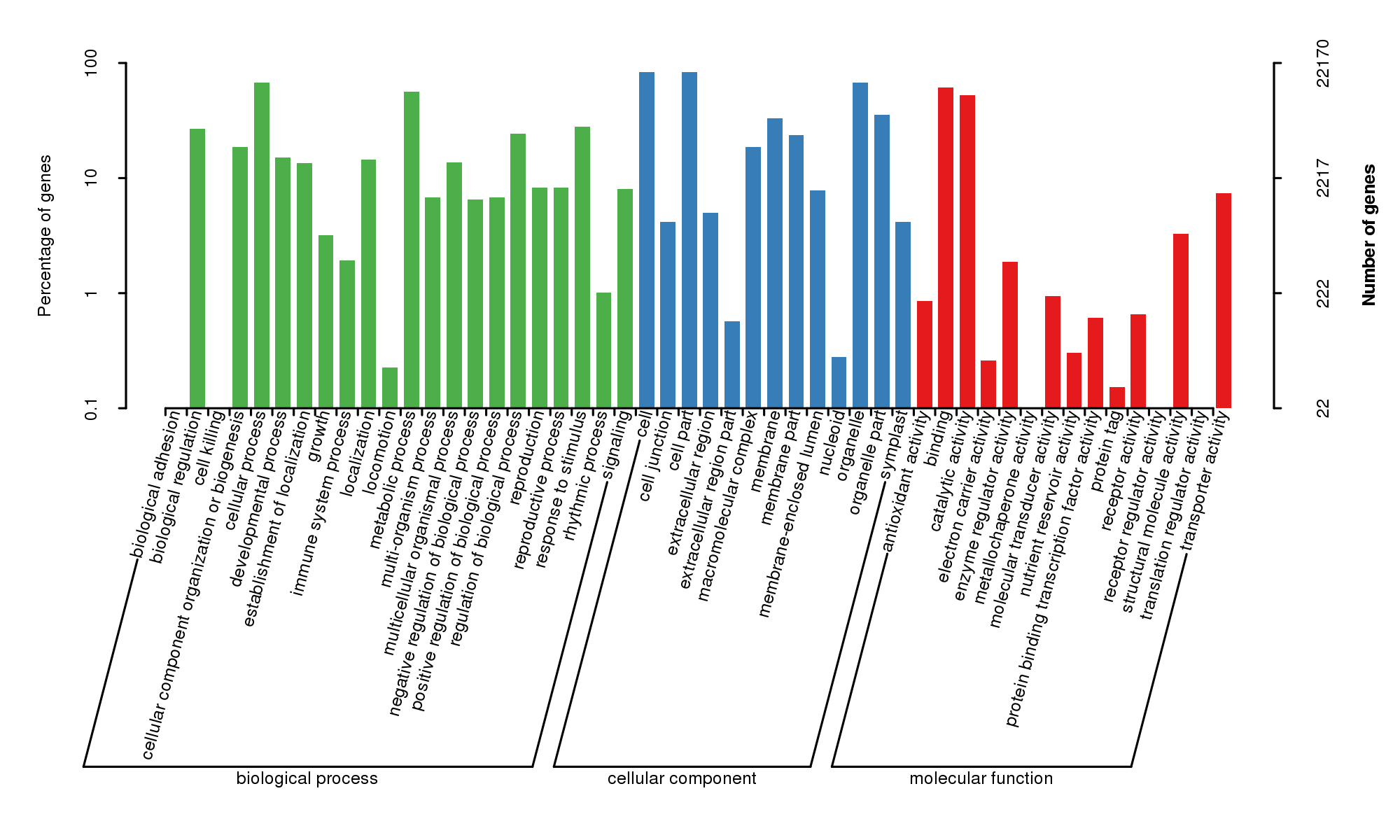


**Figure S5** **GO function annotation of *P. cyrtonema* transcriptome.**

Supplement: Supplementary file 6 — Additional file 6: Figure S5. GO function annotation of P. cyrtonema transcriptome. [file 12864_2022_8421_MOESM6_ESM.docx]

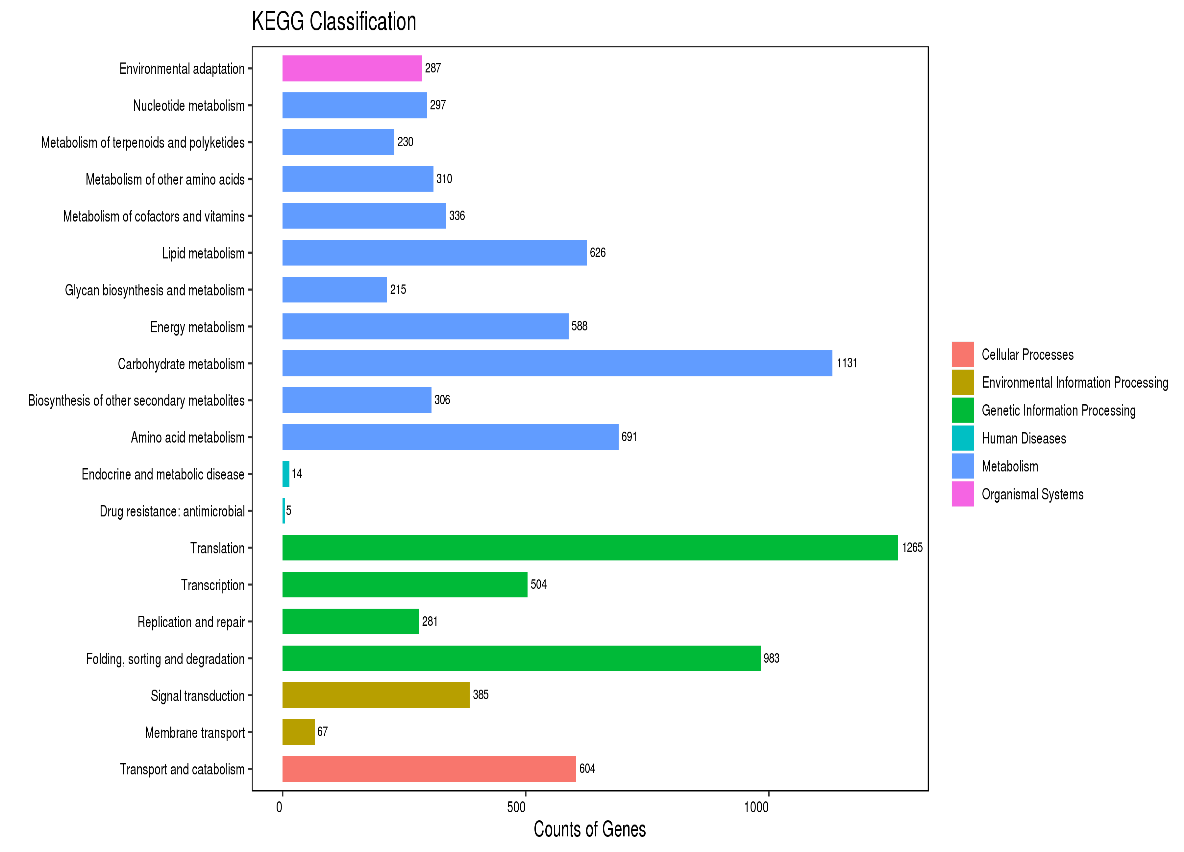


**Figure S6** **KEGG functional classifications of the annotated unigenes in *P. cyrtonema*.**

Supplement: Supplementary file 7 — Additional file 7: Figure S6. KEGG functional classifications of the annotated unigenes in P. cyrtonema. [file 12864_2022_8421_MOESM7_ESM.docx]
