# Supplementary material for: De novo assembly and analysis of Polygonatum cyrtonema Hua and identification of genes involved in polysaccharide and saponin biosynthesis
Source: BMC Genomics. 2022 Mar 10;23:195. doi: 10.1186/s12864-022-08421-y (PMC8915509; doi:10.1186/s12864-022-08421-y)
Supplement: Supplementary file 8 — Additional file 8: Table S2. KEGG annotation of all unigenes. [file 12864_2022_8421_MOESM8_ESM.docx]

**Table S2 KEGG annotation of all unigenes.**

| **class** | **Pathway definition** | **Unigene number** | **Pathway ID** |
| --- | --- | --- | --- |
| Metabolism-- Carbohydrate metabolism | Glycolysis / Gluconeogenesis | 235 | ko00010 |
| Metabolism-- Carbohydrate metabolism | Citrate cycle (TCA cycle) | 126 | ko00020 |
| Metabolism-- Carbohydrate metabolism | Pentose phosphate pathway | 104 | ko00030 |
| Metabolism-- Carbohydrate metabolism | Pentose and glucuronate interconversions | 79 | ko00040 |
| Metabolism-- Carbohydrate metabolism | Fructose and mannose metabolism | 95 | ko00051 |
| Metabolism-- Carbohydrate metabolism | Galactose metabolism | 107 | ko00052 |
| Metabolism-- Carbohydrate metabolism | Ascorbate and aldarate metabolism | 62 | ko00053 |
| Metabolism-- Lipid metabolism | Fatty acid biosynthesis | 82 | ko00061 |
| Metabolism-- Lipid metabolism | Fatty acid elongation | 48 | ko00062 |
| Metabolism-- Lipid metabolism | Fatty acid degradation | 93 | ko00071 |
| Metabolism-- Lipid metabolism | Synthesis and degradation of ketone bodies | 6 | ko00072 |
| Metabolism-- Lipid metabolism | Cutin, suberine and wax biosynthesis | 25 | ko00073 |
| Metabolism-- Lipid metabolism | Steroid biosynthesis | 43 | ko00100 |
| Metabolism-- Metabolism of cofactors and vitamins | Ubiquinone and other terpenoid-quinone biosynthesis | 55 | ko00130 |
| Metabolism-- Energy metabolism | Oxidative phosphorylation | 273 | ko00190 |
| Metabolism-- Energy metabolism | Photosynthesis | 61 | ko00195 |
| Metabolism-- Energy metabolism | Photosynthesis - antenna proteins | 21 | ko00196 |
| Metabolism-- Amino acid metabolism | Arginine biosynthesis | 59 | ko00220 |
| Metabolism-- Nucleotide metabolism | Purine metabolism | 243 | ko00230 |
| Metabolism-- Biosynthesis of other secondary metabolites | Caffeine metabolism | 8 | ko00232 |
| Metabolism-- Nucleotide metabolism | Pyrimidine metabolism | 188 | ko00240 |
| Metabolism-- Amino acid metabolism | Alanine, aspartate and glutamate metabolism | 84 | ko00250 |
| Metabolism-- Biosynthesis of other secondary metabolites | Aflatoxin biosynthesis | 17 | ko00254 |
| Metabolism-- Amino acid metabolism | Glycine, serine and threonine metabolism | 97 | ko00260 |
| Metabolism-- Biosynthesis of other secondary metabolites | Monobactam biosynthesis | 28 | ko00261 |
| Metabolism-- Amino acid metabolism | Cysteine and methionine metabolism | 185 | ko00270 |
| Metabolism-- Amino acid metabolism | Valine, leucine and isoleucine degradation | 77 | ko00280 |
| Metabolism-- Amino acid metabolism | Valine, leucine and isoleucine biosynthesis | 25 | ko00290 |
| Metabolism-- Amino acid metabolism | Lysine biosynthesis | 31 | ko00300 |
| Metabolism-- Amino acid metabolism | Lysine degradation | 50 | ko00310 |
| Metabolism-- Amino acid metabolism | Arginine and proline metabolism | 88 | ko00330 |
| Metabolism-- Amino acid metabolism | Histidine metabolism | 40 | ko00340 |
| Metabolism-- Amino acid metabolism | Tyrosine metabolism | 52 | ko00350 |
| Metabolism-- Amino acid metabolism | Phenylalanine metabolism | 54 | ko00360 |
| Metabolism-- Amino acid metabolism | Tryptophan metabolism | 39 | ko00380 |
| Metabolism-- Amino acid metabolism | Phenylalanine, tyrosine and tryptophan biosynthesis | 80 | ko00400 |
| Metabolism-- Metabolism of other amino acids | beta-Alanine metabolism | 73 | ko00410 |
| Metabolism-- Metabolism of other amino acids | Taurine and hypotaurine metabolism | 24 | ko00430 |
| Metabolism-- Metabolism of other amino acids | Phosphonate and phosphinate metabolism | 12 | ko00440 |
| Metabolism-- Metabolism of other amino acids | Selenocompound metabolism | 41 | ko00450 |
| Metabolism-- Metabolism of other amino acids | Cyanoamino acid metabolism | 58 | ko00460 |
| Metabolism-- Metabolism of other amino acids | Glutathione metabolism | 123 | ko00480 |
| Metabolism-- Carbohydrate metabolism | Starch and sucrose metabolism | 328 | ko00500 |
| Metabolism-- Glycan biosynthesis and metabolism | N-Glycan biosynthesis | 71 | ko00510 |
| Metabolism-- Glycan biosynthesis and metabolism | Other glycan degradation | 48 | ko00511 |
| Metabolism-- Glycan biosynthesis and metabolism | Various types of N-glycan biosynthesis | 55 | ko00513 |
| Metabolism-- Glycan biosynthesis and metabolism | Other types of O-glycan biosynthesis | 9 | ko00514 |
| Metabolism-- Carbohydrate metabolism | Amino sugar and nucleotide sugar metabolism | 179 | ko00520 |
| Metabolism-- Glycan biosynthesis and metabolism | Glycosaminoglycan degradation | 38 | ko00531 |
| Metabolism-- Lipid metabolism | Glycerolipid metabolism | 107 | ko00561 |
| Metabolism-- Carbohydrate metabolism | Inositol phosphate metabolism | 89 | ko00562 |
| Metabolism-- Glycan biosynthesis and metabolism | Glycosylphosphatidylinositol(GPI)-anchor biosynthesis | 36 | ko00563 |
| Metabolism-- Lipid metabolism | Glycerophospholipid metabolism | 147 | ko00564 |
| Metabolism-- Lipid metabolism | Ether lipid metabolism | 38 | ko00565 |
| Metabolism-- Lipid metabolism | Arachidonic acid metabolism | 23 | ko00590 |
| Metabolism-- Lipid metabolism | Linoleic acid metabolism | 31 | ko00591 |
| Metabolism-- Lipid metabolism | alpha-Linolenic acid metabolism | 102 | ko00592 |
| Metabolism-- Lipid metabolism | Sphingolipid metabolism | 68 | ko00600 |
| Metabolism-- Glycan biosynthesis and metabolism | Glycosphingolipid biosynthesis - globo series | 15 | ko00603 |
| Metabolism-- Glycan biosynthesis and metabolism | Glycosphingolipid biosynthesis - ganglio series | 6 | ko00604 |
| Metabolism-- Carbohydrate metabolism | Pyruvate metabolism | 155 | ko00620 |
| Metabolism-- Carbohydrate metabolism | Glyoxylate and dicarboxylate metabolism | 140 | ko00630 |
| Metabolism-- Carbohydrate metabolism | Propanoate metabolism | 78 | ko00640 |
| Metabolism-- Carbohydrate metabolism | Butanoate metabolism | 28 | ko00650 |
| Metabolism-- Carbohydrate metabolism | C5-Branched dibasic acid metabolism | 8 | ko00660 |
| Metabolism-- Metabolism of cofactors and vitamins | One carbon pool by folate | 24 | ko00670 |
| Metabolism-- Energy metabolism | Carbon fixation in photosynthetic organisms | 143 | ko00710 |
| Metabolism-- Metabolism of cofactors and vitamins | Thiamine metabolism | 16 | ko00730 |
| Metabolism-- Metabolism of cofactors and vitamins | Riboflavin metabolism | 18 | ko00740 |
| Metabolism-- Metabolism of cofactors and vitamins | Vitamin B6 metabolism | 13 | ko00750 |
| Metabolism-- Metabolism of cofactors and vitamins | Nicotinate and nicotinamide metabolism | 34 | ko00760 |
| Metabolism-- Metabolism of cofactors and vitamins | Pantothenate and CoA biosynthesis | 38 | ko00770 |
| Metabolism-- Metabolism of cofactors and vitamins | Biotin metabolism | 29 | ko00780 |
| Metabolism-- Metabolism of cofactors and vitamins | Lipoic acid metabolism | 7 | ko00785 |
| Metabolism-- Metabolism of cofactors and vitamins | Folate biosynthesis | 43 | ko00790 |
| Metabolism-- Metabolism of cofactors and vitamins | Porphyrin and chlorophyll metabolism | 63 | ko00860 |
| Metabolism-- Metabolism of terpenoids and polyketides | Terpenoid backbone biosynthesis | 87 | ko00900 |
| Metabolism-- Metabolism of terpenoids and polyketides | Monoterpenoid biosynthesis | 11 | ko00902 |
| Metabolism-- Metabolism of terpenoids and polyketides | Limonene and pinene degradation | 14 | ko00903 |
| Metabolism-- Metabolism of terpenoids and polyketides | Diterpenoid biosynthesis | 24 | ko00904 |
| Metabolism-- Metabolism of terpenoids and polyketides | Brassinosteroid biosynthesis | 16 | ko00905 |
| Metabolism-- Metabolism of terpenoids and polyketides | Carotenoid biosynthesis | 46 | ko00906 |
| Metabolism-- Metabolism of terpenoids and polyketides | Zeatin biosynthesis | 25 | ko00908 |
| Metabolism-- Metabolism of terpenoids and polyketides | Sesquiterpenoid and triterpenoid biosynthesis | 8 | ko00909 |
| Metabolism-- Energy metabolism | Nitrogen metabolism | 47 | ko00910 |
| Metabolism-- Energy metabolism | Sulfur metabolism | 52 | ko00920 |
| Metabolism-- Biosynthesis of other secondary metabolites | Phenylpropanoid biosynthesis | 195 | ko00940 |
| Metabolism-- Biosynthesis of other secondary metabolites | Flavonoid biosynthesis | 41 | ko00941 |
| Metabolism-- Biosynthesis of other secondary metabolites | Flavone and flavonol biosynthesis | 7 | ko00944 |
| Metabolism-- Biosynthesis of other secondary metabolites | Stilbenoid, diarylheptanoid and gingerol biosynthesis | 26 | ko00945 |
| Metabolism-- Biosynthesis of other secondary metabolites | Isoquinoline alkaloid biosynthesis | 21 | ko00950 |
| Metabolism-- Biosynthesis of other secondary metabolites | Tropane, piperidine and pyridine alkaloid biosynthesis | 32 | ko00960 |
| Metabolism-- Biosynthesis of other secondary metabolites | Betalain biosynthesis | 3 | ko00965 |
| Genetic Information Processing--Translation | Aminoacyl-tRNA biosynthesis | 120 | ko00970 |
| Metabolism-- Lipid metabolism | Biosynthesis of unsaturated fatty acids | 56 | ko01040 |
| Human Diseases-- Drug resistance: antimicrobial | Vancomycin resistance | 5 | ko01502 |
| Environmental Information Processing-- Membrane transport | ABC transporters | 67 | ko02010 |
| Genetic Information Processing-- Translation | Ribosome biogenesis in eukaryotes | 149 | ko03008 |
| Genetic Information Processing—Translation | Ribosome | 548 | ko03010 |
| Genetic Information Processing-- Translation | RNA transport | 313 | ko03013 |
| Genetic Information Processing-- Translation | mRNA surveillance pathway | 220 | ko03015 |
| Genetic Information Processing-- Folding, sorting and degradation | RNA degradation | 212 | ko03018 |
| Genetic Information Processing-- Transcription | RNA polymerase | 62 | ko03020 |
| Genetic Information Processing-- Transcription | Basal transcription factors | 66 | ko03022 |
| Genetic Information Processing-- Replication and repair | DNA replication | 136 | ko03030 |
| Genetic Information Processing-- Transcription | Spliceosome | 376 | ko03040 |
| Genetic Information Processing-- Folding, sorting and degradation | Proteasome | 95 | ko03050 |
| Genetic Information Processing-- Folding, sorting and degradation | Protein export | 87 | ko03060 |
| Genetic Information Processing-- Replication and repair | Base excision repair | 60 | ko03410 |
| Genetic Information Processing-- Replication and repair | Nucleotide excision repair | 115 | ko03420 |
| Genetic Information Processing-- Replication and repair | Mismatch repair | 89 | ko03430 |
| Genetic Information Processing-- Replication and repair | Homologous recombination | 98 | ko03440 |
| Genetic Information Processing-- Replication and repair | Non-homologous end-joining | 12 | ko03450 |
| Environmental Information Processing-- Signal transduction | Phosphatidylinositol signaling system | 92 | ko04070 |
| Environmental Information Processing-- Signal transduction | Plant hormone signal transduction | 293 | ko04075 |
| Genetic Information Processing-- Folding, sorting and degradation | Ubiquitin mediated proteolysis | 216 | ko04120 |
| Genetic Information Processing-- Folding, sorting and degradation | Sulfur relay system | 17 | ko04122 |
| Genetic Information Processing-- Folding, sorting and degradation | SNARE interactions in vesicular transport | 74 | ko04130 |
| Genetic Information Processing-- Folding, sorting and degradation | Protein processing in endoplasmic reticulum | 357 | ko04141 |
| Cellular Processes-- Transport and catabolism | Endocytosis | 302 | ko04144 |
| Cellular Processes-- Transport and catabolism | Phagosome | 174 | ko04145 |
| Cellular Processes-- Transport and catabolism | Peroxisome | 137 | ko04146 |
| Organismal Systems-- Environmental adaptation | Plant-pathogen interaction | 229 | ko04626 |
| Organismal Systems-- Environmental adaptation | Circadian rhythm - plant | 58 | ko04712 |
| Human Diseases-- Endocrine and metabolic disease | AGE-RAGE signaling pathway in diabetic complications | 14 | ko04933 |
