# Supplementary material for: De novo assembly and analysis of Polygonatum cyrtonema Hua and identification of genes involved in polysaccharide and saponin biosynthesis
Source: BMC Genomics. 2022 Mar 10;23:195. doi: 10.1186/s12864-022-08421-y (PMC8915509; doi:10.1186/s12864-022-08421-y)
Supplement: Supplementary file 9 — Additional file 9: Table S3. The numerical values of error bar. [file 12864_2022_8421_MOESM9_ESM.docx]

**Table S3** **The numerical values of error bar.**

| **RNA-seq** | **gene** | **One-year** | **Two-year** | **Three-year** | **Four-year** |
| --- | --- | --- | --- | --- | --- |
|  | SacA | 69.82±0.26 | 659.82±6.50 | 1133.95±29.47 | 274.31±2.56 |
|  | HK | 22.50±1.21 | 35.20±3.39 | 46.70±3.56 | 25.60±3.89 |
|  | ScrK | 329.9±2.74 | 79.4±0.87 | 69.8±2.12 | 47.6±2.46 |
|  | MPI | 21.8±1.23 | 39.4±1.31 | 80.5±3.06 | 40.4±0.80 |
|  | PGM | 67.3±1.42 | 89.4±1.85 | 91.0±2.27 | 65.5±1.00 |
|  | AXS | 21.2±2.26 | 44.2±0.12 | 67.9±2.74 | 25.4±0.43 |
|  | HGMS | 22.23±0.98 | 33.64±0.53 | 63.46±4.59 | 22.98±1.67 |
|  | MVD | 32.49±1.99 | 44.46±2.79 | 61.88±2.20 | 34.04±1.65 |
|  | IDI | 33.33±0.98 | 49.67±3.05 | 46.89±2.22 | 41.98±2.11 |
|  | ispH | 24.25±0.74 | 25.93±0.94 | 57.19±2.69 | 20.43±0.76 |
|  | FPS | 18.29±1.16 | 35.46±2.28 | 48.20±0.86 | 27.25±0.73 |
|  | SS | 24.19±0.77 | 33.78±1.77 | 36.55±1.58 | 41.45±0.73 |
| **qRT-PCR** | gene | One-year | Two-year | Three-year | Four-year |
|  | SacA | 1.00±0.05 | 10.21±0.20 | 19.35±0.32 | 3.91±0.15 |
|  | HK | 1.00±0.03 | 1.48±0.05 | 1.79±0.05 | 1.36±0.06 |
|  | ScrK | 4.84±0.04 | 1.32±0.02 | 1.17±0.07 | 1.00±0.02 |
|  | MPI | 1.00±0.01 | 1.66±0.08 | 3.42±0.15 | 2.25±0.08 |
|  | PGM | 1.07±0.08 | 1.18±0.07 | 1.42±0.06 | 1.00±0.01 |
|  | AXS | 1.00±0.03 | 2.30±0.01 | 2.73±0.01 | 1.34±0.11 |
|  | HGMS | 6.87±0.21 | 13.14±0.50 | 11.26±1.53 | 1.00±0.08 |
|  | MVD | 1.11±0.11 | 1.27±0.05 | 1.49±0.05 | 1.00±0.01 |
|  | IDI | 1.00±0.01 | 1.29±0.04 | 1.06±0.11 | 0.91±0.43 |
|  | ispH | 1.25±0.02 | 1.38±0.01 | 2.66±0.08 | 1.00±0.06 |
|  | FPS | 1.00±0.03 | 1.74±0.04 | 2.43±0.11 | 1.55±0.10 |
|  | SS | 1.02±0.24 | 1.37±0.04 | 1.49±0.04 | 1.73±0.15 |
