# Supplementary material for: De novo assembly and analysis of Polygonatum cyrtonema Hua and identification of genes involved in polysaccharide and saponin biosynthesis
Source: BMC Genomics. 2022 Mar 10;23:195. doi: 10.1186/s12864-022-08421-y (PMC8915509; doi:10.1186/s12864-022-08421-y)
Supplement: Supplementary file 10 — Additional file 10: Table S4. RNA information of different samples. [file 12864_2022_8421_MOESM10_ESM.docx]

**Table S4** **RNA information of different samples.**

| Sample | Concentration (ng/μL) | Total amount (μg) | OD260/280 | RIN | 28S/18S |
| --- | --- | --- | --- | --- | --- |
| One-year-1 | 302.8 | 3.03 | 2.16 | 10 | 1.6 |
| One-year-2 | 310.6 | 3.11 | 2.15 | 10 | 1.7 |
| One-year-3 | 300.3 | 3.00 | 2.16 | 10 | 1.7 |
| Two-year-1 | 276.9 | 2.77 | 2.17 | 10 | 1.7 |
| Two-year-2 | 268.9 | 2.69 | 2.16 | 10 | 1.6 |
| Two-year-3 | 273.9 | 2.74 | 2.16 | 10 | 1.6 |
| Three-year-1 | 162.3 | 1.62 | 2.17 | 10 | 1.9 |
| Three-year-2 | 159.9 | 1.60 | 2.17 | 10 | 1.9 |
| Three-year-3 | 164.5 | 1.65 | 2.18 | 10 | 2.0 |
| Four-year-1 | 250.8 | 2.51 | 2.17 | 10 | 1.9 |
| Four-year-2 | 246.8 | 2.47 | 2.18 | 10 | 1.9 |
| Four-year-3 | 249.9 | 2.50 | 2.16 | 10 | 1.9 |
