# Supplementary material for: De novo assembly and analysis of Polygonatum cyrtonema Hua and identification of genes involved in polysaccharide and saponin biosynthesis
Source: BMC Genomics. 2022 Mar 10;23:195. doi: 10.1186/s12864-022-08421-y (PMC8915509; doi:10.1186/s12864-022-08421-y)
Supplement: Supplementary file 11 — Additional file 11: Table S5. Gene descriptions and primers used for qRT-PCR. [file 12864_2022_8421_MOESM11_ESM.docx]

**Table S5** **Gene descriptions and primers used for qRT-PCR.**

| Genes | Amplicon Size （bp） | Primer pairs | TM value |
| --- | --- | --- | --- |
| TRINITY_DN27092_c0_g5_i1_1 | 107 | 5'-TGAAATCGTGAAGGAAGTCTCT-3' | 58.06 |
|  |  | 5'-TCTCAATCATGTTGTCACCTTC-3' | 57.64 |
| TRINITY_DN30222_c0_g1_i2_3 | 150 | 5'-AACCAAAGACTTCCTCTCCTTC-3' | 58.01 |
|  |  | 5'-CCTGGACTAGTCACCAAACTGT-3' | 55.24 |
| TRINITY_DN31573_c0_g2_i6_1 | 121 | 5'-CTTTACTTTCTCGTTCCCAGTG-3' | 58.06 |
|  |  | 5'-CTTGGTCAATTCAGCAACTACA-3' | 57.96 |
| TRINITY_DN29996_c0_g2_i6_1 | 111 | 5'-TCTTCTCTCGTACGATCCAAAC-3' | 58.46 |
|  |  | 5'-TCGCTGATCTTTATGATGTCTG-3' | 58.00 |
| TRINITY_DN25745_c0_g1_i1_1 | 122 | 5'-CTGAAATTAAAGAGCTGGTTGG-3' | 58.08 |
|  |  | 5'-GGCCGACATAAACTTAGTGAAG-3' | 57.97 |
| TRINITY_DN33755_c0_g1_i1_1 | 146 | 5'-ATCCTGTTGATGGTTCTGTCTC-3' | 58.10 |
|  |  | 5'-CGAAGAGTCCTTCTCATATTGC-3' | 58.10 |
| TRINITY_DN33560_c1_g1_i11_1 | 119 | 5'-GGTTTCTGGTGATGTATGGTTT-3' | 57.84 |
|  |  | 5'-TCACCTTGTTCTCGAGTACCTT-3' | 57.99 |
| TRINITY_DN30891_c0_g1_i11_3 | 118 | 5'- GTCACAGTAAAGCAACTTGCAG-3' | 57.78 |
|  |  | 5'-ACCCTTCACCATAGAACTCCTT-3' | 58.15 |
| TRINITY_DN19479_c0_g1_i1_3 | 126 | 5'-GTACATTGACCCAAGCTTCACT-3' | 58.25 |
|  |  | 5'-AGAAGCTCAGGGAACTCTTTCT-3' | 57.92 |
| TRINITY_DN28704_c0_g1_i3_1 | 136 | 5'-GGTGATGAAAGCTACCAAAGTC-3' | 57.87 |
|  |  | 5'-CATAAAGGGATGCCGTATACAT-3' | 57.93 |
| TRINITY_DN32691_c1_g1_i2_2 | 130 | 5'-CAGTGGTAAGTTCACGACAGAA-3' | 57.94 |
|  |  | 5'-GCTTCTTCCATTTGCAGTATTC-3' | 58.03 |
| TRINITY_DN27696_c0_g1_i6_2 | 103 | 5'-GCTTGATTACCTGCTTTTCATC-3' | 58.03 |
|  |  | 5'- TAGCTCCTTCAATTGATCCCTA-3' | 57.97 |
| TRINITY_DN24041_c0_g1_i1_3 | 118 | 5'-GAAATCATCCATAATCCCACTG-3' | 58.25 |
|  |  | 5'-AAACCACTACATCACCCTCATC-3' | 57.91 |
| TRINITY_DN27061_c0_g1_i1_3 | 105 | 5'-TCCAAGAGGAAGAGGAGTACAA-3' | 58.06 |
|  |  | 5'-CAGCATGTACTTCTTGAAATCG-3' | 57.60 |
| TRINITY_DN31531_c0_g1_i3_2 | 132 | 5'-AAGGAGAACAAGACCTGTCAAA-3' | 57.96 |
|  |  | 5'-AGTTCTCCAACTTTTCACCAGA-3' | 57.96 |
| TRINITY_DN31356_c0_g1_i3_1 | 136 | 5'-AGGTTTTCAGAGGAGTTGTGAA-3' | 57.96 |
|  |  | 5'-TCACTCTTATCCACCTTCGACT-3' | 57.94 |
| TRINITY_DN28778_c0_g3_i2_1 | 122 | 5'-AAGCAACCATCACATACAACTG-3' | 57.69 |
|  |  | 5'-CAGGGAACTGTAACTACCTTGC-3' | 57.95 |
